# Supplementary material for: The Molecular Mechanism of Farnesoid X Receptor Alleviating Glucose Intolerance in Turbot (Scophthalmus maximus)
Source: Cells. 2024 Nov 23;13(23):1949. doi: 10.3390/cells13231949 (PMC11640315; doi:10.3390/cells13231949)
Supplement: Supplementary file 1 [file cells-13-01949-s001.zip › cells-3271183-supplementary.pdf]

**Table S1 Sequences of the gene cloning and qPCR primers**

| Function | Primers | Sequence (5’-3’) | GenBank Accession Number |
|---|---|---|---|
| Gene clone | FXR-F | ATGAATGAGTGGGTGGGCCCT | XM_118315928 |
| | FXR-R | AATGAGGTGCAGGGTATAGAG | |
| Sequences of the qPCR primers | qFXR-F | AGGCGGCGGACAACACAGAA | XM_118315928 |
| | qFXR-R | AGCGCCTGCTGCTCTTTGGT | |
| | SHP1-F | AAACCGAGTCGAGGCAACTT | XM_035630214.2 |
| | SHP1-R | TTAACCAGAACCCCCGAAGC | |
| | *foxo1*-F | CCGTACCCATTGGTGTTGAGG | MF945551 |
| | *foxo1*-R | CCGCCGTACAGCAGCAGTCA | |
| | *gk*-F | CGACACGAGGACATTGACAAG | JX678944 |
| | *gk*-R | CCAACAATCATCCCGACTTCAC | |
| | *pk*-F | TGGATACGCTGAAGGAGATG | DQ848903 |
| | *pk*-R | ACGCACGTTCTTGATGGTC | |
| | *cpepck*-F | GTGTTTGTTGGAGCAGCCATGAG | KC149517 |
| | *cpepck*-R | GCTCTTGCGGAACCAGTTGACG | |
| | *mpepck*-F | CTGGTCTACGAGTCGTTCAAC | KC149516 |
| | *mpepck*-R | GGTAGTCGCCAAAGTTGTAGC | |
| | *g6pase1*-F | CACGAGACGGCTCATTATGC | KC184131 |
| | *g6pase1*-R | CTTTGCTGCTGGATTTCTTGC | |
| | *g6pase2*-F | GGCTCAACTTGGTCCTGAAATG | KC184132 |
| | *g6pase2*-R | GCGCTGTTACCATCACATACCAG | |
| | *β-actin*-F | GTAGGTGATGAAGCCCAGAGCA | NC_061531.1 |
| | *β-actin*-R | CTGGGTCATCTTCTCCCTGT | |
| | *rps4*-F | CAACATCTTCGTCATCGGCAAGG | NC_061526.1 |
| | *rps4-R* | ATTGAACCAGCCTCAGTGTTTAGC | |

**Table S2 Sequences of the plasmid construction primers**

| Functions | Primers | Sequence (5'-3') |
|---|---|---|
| Expressing plasmids construction | CMV-RXR-HA-F | ggcggatccaagcttctgcagATGGAAACCAAACCGTTCCTG |
| | CMV-RXR-HA-R | gacgatatcgaattcctgcagTGTCATCTGATGCGGTGCTTC |
| | CMV-FXR-flag-F | ggcggatccaagcttctgcagATGAATGAGTGGGTGGGCCC |
| | CMV-FXR-flag-R | gacgatatcgaattcctgcagCTCCACGTCCCATATCTCACAGA |
| | FXR-GFP-F | cttggtaccgagctcggatccATGAATGAGTGGGTGGGCCC |
| | FXR-GFP -R | atggtggcgaccggtggatccCTCCACGTCCCATATCTCACAGA |
| | pcDNA3.1-SHP-F | agtggatccgagctcggtaccATGGATAACGGATGTCATTGTTCG |
| | pcDNA3.1-SHP-R | ctagcgtttaaacttaagcttCTATCTGCAGAAGAGCATGTCGAC |
| Report plasmids construction | pGL-SHP- F | atctgcgatctaagtaagcttGCACAAGGAGAGCAATGTATGGG |
| | pGL-SHP- R | cagtaccggaatgccaagcttAAAGCCTATCCGAGCTGAATCC |
| | pGL-mPEPCK1-F | atttctctatcgataggtaccTCCAACTTGAATTCCTCTTTTTTAAA |
| | pGL-mPEPCK1-R | cagtaccggaatgccaagcttGTTCCCTGCTGATCTGAACAACA |
| | pGL- cPEPCK1-F | atttctctatcgataggtaccCTACAGTATCTCCTACTGTCCAACCTG |
| | pGL- cPEPCK1-R | cagtaccggaatgccaagcttCTCAGGCCCCGGGTTTTC |
| | pGL-PK-F | atttctctatcgataggtaccCTCTTTGAATTGTGCCATTATATACTGC |
| | pGL-PK-R | cagtaccggaatgccaagcttTCCACTACTTGTCATGTGCGATG |
| | pGL-G6PASE1-F | atttctctatcgataggtaccTGCTGGCGCTGTAGATCCA |
| | pGL-G6PASE1-R | cagtaccggaatgccaagcttGCCTCCCAAACTCCCCTGA |
| | pGL-G6PASE 2-F | atttctctatcgataggtaccTACCATCACATACCAGACACCCG |
| | pGL-G6PASE 2-R | cagtaccggaatgccaagcttCAGCTGGTTGGTTATATACCAGAGG |
| siSHP sequences for knocking down | siSHP-NC-F | UUCUCCGAACGUGUCACGUTT |
| | siSHP-NC-R | ACGUGACACGUUCGGAGAATT |
| | siSHP-171-F | GCCAGUUGAGAUUUGCAAATT |
| | siSHP-171-R | UUUGCAAAUCUCAACUGGCTT |
| | siSHP-495-F | GGAGUAUGCAUACCUCAAATT |
| | siSHP-495-R | UUUGAGGUAUGCAUACUCCTT |
| | siSHP-532-F | CCAGAUGUUCCAGAUUUAATT |
| | siSHP-532-R | UUAAAUCUGGAACAUCUGGTT |
